# Written exposure therapy as treatment for comorbid PTSD and SUD: a systematic review and meta-analysis

**DOI:** 10.1007/s11920-026-01724-x

**Published:** 2026-11-07

**Authors:** Tooba Fatima, Sarah Catchlove, Satish Melwani, Bosco Rowland, Shalini Arunogiri

**Affiliations:** 1 Eastern Health Clinical School, Faculty of Medicine, Nursing and Health Sciences, Monash University, Melbourne, Australia; 2 Turning Point, Eastern Health, Melbourne, Australia

**Keywords:** Post-Traumatic Stress Disorder (PTSD), Substance Use Disorder (SUD), Comorbid, Written Exposure Therapy (WET), Integrated treatment

## Abstract

**Purpose of review:**

This study aimed to evaluate the effectiveness, feasibility, and acceptability of Written Exposure Therapy (WET) when it is used as a component in integrated treatment of comorbid PTSD and Substance Use Disorder (SUD). A systematic review and meta-analysis was used to synthesise foundational knowledge regarding WET.

**Recent findings:**

Twelve studies were included and analysed in the review. Random-effects meta-analysis of six studies showed large overall pre/post decrease in PTSD severity (Hedges’ *g* = 1.18, 95% CI [0.68, 1.72], *p* < 0.001). Rate of retention (69.03%) was higher than typically seen in integrated prolonged exposure treatments for PTSD-SUD. The majority of participants and providers found WET feasible and acceptable. A minority of participants found concurrent treatment for both disorders burdensome.

**Summary:**

Findings suggest that can be an effective component in integrated treatment of PTSD-SUD, owing to its efficacy, brevity, and low-burden of integration. Limitations of the current findings include small samples, lack of comparator groups, and limited information regarding treatment components and change in SUD.

**Supplementary Information:**

The online version contains supplementary material available at https://doi.org/10.1007/s11920-026-01724-x.

## Introduction

Post Traumatic Stress Disorder (PTSD) and Substance Use Disorder (SUD) are significant mental health concerns that are frequently comorbid. Prevalence of this comorbidity ranges from 25–49% in the general population, and is typically higher in clinical populations [1–3]. Patients with comorbid PTSD-SUD experience a more severe, complex, costly, and chronic course of illness compared to patients with either disorder alone. Unfortunately, both disorders maintain each other, impede recovery for either condition, and are more difficult to treat when comorbid [1, 4–9].

PTSD and traumatic exposure exert significant influence over the impacts and outcomes of PTSD-SUD. Substance use is more common in individuals who report trauma exposure or meet criteria for PTSD, with a higher number of traumatic events becoming increasingly predictive of SUD severity [10, 11]. PTSD severity predicts faster relapse following SUD treatment, with patients endorsing elevated PTSD symptoms prior to and immediately following relapse [7, 12, 13]. Finally, a reduction in PTSD severity accounts for a greater and more sustained reduction in SUD, while a reduction in SUD severity does not account for a similar or greater reduction in PTSD [7, 8, 12, 14].

### Treatment of comorbid PTSD-SUD

Growing evidence suggests that exposure-based integrated interventions are safe, tolerable, and more effective than non-exposure-based interventions in the treatment of PTSD-SUD, without exacerbating or compromising outcomes for SUD [12, 14–20]. However, existing treatments have inherent constraints that limit their accessibility and retention. Integrated treatments are typically longer than treatment for either disorder [21], requiring between 12–15 sessions that each last 60–90 min [20, 22, 23]. Most clinicians manage high clinical workloads, and are typically trained for either PTSD or SUD but not both. The burden of resources and time required by specialized integrated treatments is quite high [4, 24]. Finally, studies show that less than 40% of participants receive an adequate dose of exposure before dropping out of integrated treatment, and more than 40% leave prior to completion [25–28].

In this landscape, Written Exposure Therapy (WET) is emerging as a modality of interest. WET is a brief therapy utilising exposure through writing, across 5–7 60-min sessions, with no between-session homework [29]. It has been established as an effective treatment for PTSD across a variety of demographic and cultural contexts [30] and is shown to be non-inferior to existing exposure-based therapies [31, 32]. Recent literature has started to explore its use for the treatment of PTSD-SUD, as it offers several potential advantages. Its brevity can allow for easier integration into existing SUD treatment programs. Its highly structured delivery and low training burden may make it more feasible for clinicians. And in its less cognitively-demanding nature, it may also be more accessible to a broader range of clients. Finally, by both frontloading the exposure in the first session and via writing, WET may also reduce treatment dropout.

To date, only exploratory and pilot studies with small samples have explored the potential of WET as a treatment for PTSD-SUD. However, no systematic collation of WET’s effectiveness for PTSD-SUD treatment exists in the current literature. Therefore, we conducted a systematic review and meta-analysis of the international literature to:

1. Examine changes in PTSD symptoms following Written Exposure Therapy (WET) among individuals with comorbid PTSD and substance use disorders (SUD).
2. Evaluate preliminary evidence regarding the impact of WET integrated within SUD treatment settings on substance use–related outcomes..
3. Assess the feasibility and acceptability of WET as a component of integrated treatment for PTSD-SUD.

## Method

The Preferred Reporting Items for Systematic Reviews and Meta-Analysis guidelines were followed for conducting this review (PRISMA 2020) [33]. The review was registered with the Prospero International Prospective Register for Systematic Reviews (PROSPERO; ID CRD42024602565) [34]. Studies that implement or plan to implement WET as treatment for adults presenting with co-morbid PTSD/trauma and SUD were included. Detailed inclusion criteria and search terms used are detailed in Appendix A. The initial search was conducted in November 2024 and included publications from January 2000 to October 2024. Studies short-listed for inclusion were screened by two reviewers and any disputes were resolved by discussion among all three reviewers.

### Data extraction and analysis

A data extraction template was created in Covidence. Data were extracted by two reviewers regarding the publication, sample size and demographic characteristics, retention, any comparators or control treatments, psychometric measures, data analysis strategy, reported outcomes, conclusions, and limitations of the study.

A risk of bias assessment (RoB) was conducted for studies that reported quantitative outcomes for change in PTSD or SUD, as these were considered most relevant to the goals of this review. The Cochrane RoB in Non-Randomised Studies – of Interventions Version 2 (ROBINS-I-V2) [35] was used to assess pilot studies, and the RoB 2 [36] was used to assess RCTs. The RoB was completed independently by two reviewers, and discrepancies were resolved with discussion among all three reviewers.

A meta-analysis was considered where results were reported by 3 or more studies for psychometric tools that measure change in PTSD or SUD. Only the PCL-5 met these criteria, results for which were reported by six studies. Standardized mean change effect sizes (Hedges’ *g*) were calculated using pre- and post-treatment PTSD symptom scores among participants receiving WET. Change-score standard deviations were derived from reported baseline and follow-up standard deviations using an assumed pre–post correlation of *r* = 0.50 when change-score standard deviations were not reported. Small-sample bias was corrected using Hedges’ adjustment. Random-effects meta-analysis models were fitted using restricted maximum likelihood estimation (REML). Statistical heterogeneity was assessed using Cochran’s *Q*, *I*^2^ and τ^2^ statistics. Effect sizes were coded such that positive values reflected improvement in PTSD symptoms from pre- to post-treatment. Egger’s regression test was conducted to examine potential small-study effects and publication bias.

Qualitative findings were sparse, varied, and distributed across different studies. A Narrative Synthesis approach was used to consolidate findings regarding change in PTSD and SUD, and feasibility and acceptability of WET. Reviewers followed guidelines for using narrative synthesis for systematic reviews outlined by Economic and Social Council Methods [37].

## Results

**Fig. 1:**
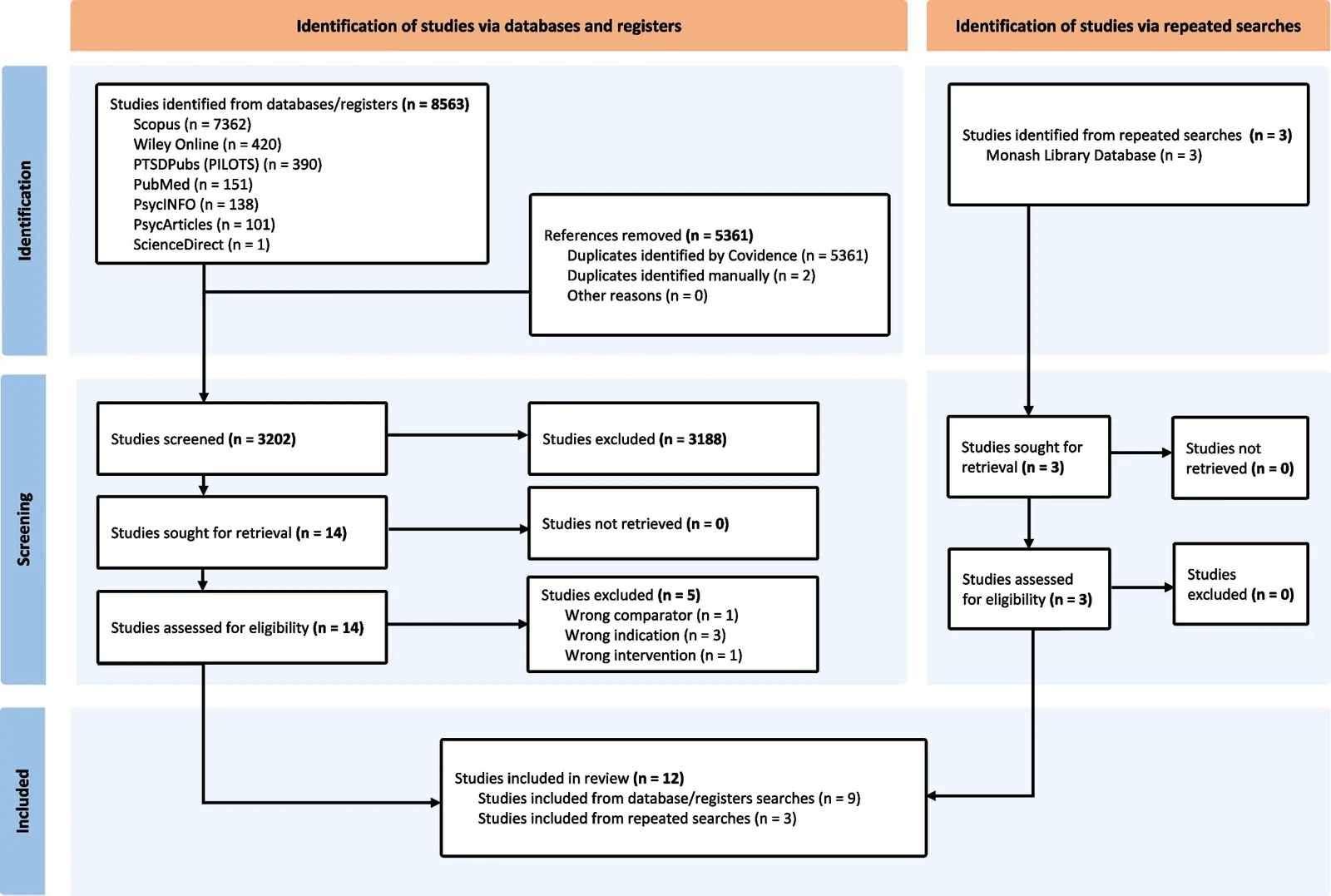
PRISMA flowchart of study identification, screening, and inclusion from database searches and repeated searches

The preliminary search resulted in the identification of 8563 articles. Duplicates (*n* = 5361) were removed using Covidence software followed by manual screening by a reviewer. 3202 studies were screened at the title and abstract level, and 14 full-text studies were assessed for eligibility. Following inclusion criteria, 9 articles were included in the review at this stage. Given the small number of studies, searches were repeated in November 2025 and May 2026 via the Monash Library Database, with the goal of finding new publications. Three additional studies met inclusion criteria and included in the review. A total of 12 studies were included and analysed in the review. Figure 1 shows a PRISMA flowchart of the identification, screening, and inclusion of studies from the initial database search and repeated searches. Table 1 summarises key information extracted from each study. An identifier number (#1–12) is assigned to each study for ease of reference.

**Table 1:** Summary of studies, identifier numbers, study setting, population, follow-up period following intervention, substances of choice, use of WET, and comparator conditions if any

	**Study (Year)**	**#**	**Setting**	**Population**	**Primary Substance(s) Used**	**Use of WET**	**Control or Comparator**	**Follow-up Period**
**Quantitative Studies**	Nillni (2023)	1	Specialist Obstetrics-Addiction clinic	Pregnant women	Any or poly-substance	Standard WET	None	6 months post-partum
	Schacht (2023)	2	Residential SUD Treatment	Patients in treatment	Any or poly-substance	Standard WET	None	Nil
	Van Doren (2025)	3	Residential SUD Treatment	Veterans	Any or poly-substance	Twice-weekly Group format	None	Nil
	Hahn (2026)	4	Study clinic	Females with recent sexual assault	Alcohol	WET + CBT for AUD	None	1 month
	Shaw (2025)	5	Residential SUD Treatment	Veterans	Any or poly-substance	Standard WET	None	1 month after discharge
	Schacht (2026)	6	Residential SUD Treatment	Patients in treatment	Any or poly-substance	Standard WET + TAU	TAU only	Nil
**Qualitative Studies**	Valentine (2023 a)	7	Specialist Obstetrics-Addiction clinic	Treatment providers	Any or poly-substance	Standard WET	None	Nil
	Valentine (2023 b)	8	Specialist Obstetrics-Addiction clinic	Pregnant Women	Any or poly-substance	Standard WET	None	Nil
	Schumacher (2023)	9	Residential SUD Treatment	Patients in treatment	Any or poly-substance	Twice-weekly Group Format	None	Nil
	Hahn (2025 b)	10	Study clinic	Females with recent sexual assault	Cannabis	WET + CBT for CUD	None	1 month
**RCT Protocols**	Hahn (2023)	11	Study clinic	Females with recent sexual assault	Alcohol	WET + CBT for AUD	Standard WET	5 weeks, 1-, 3-, and 6-months
	Meshberg-Cohen (2024)	12	Residential SUD Treatment	Veterans	Any or poly-substance	Standard WET	Neutral Topic Writing + TAU	8 weeks, 12 weeks

^#^Denotes identifier number assigned to each study for ease of reference throughout this paper

*SUD* Substance Use Disorder, *AUD* Alcohol Use Disorder, *CUD* Cannabis Use Disorder, *CBT* Cognitive Behavioural Therapy, *WET* Written Exposure Therapy, *TAU* Treatment as Usual

### Study descriptions

All twelve studies were based in the United States. Six studies reported quantitative findings, five from single-arm pilots (#1–5) and one from a feasibility RCT (#6). Nillni et al. [38] (#1) provided WET to pregnant women in a specialised addictions-obstetrics outpatient clinic. Schacht et al. [39] (#2) and Shaw et al. [40] (#5)conducted non-randomised feasibility pilots of WET in residential SUD treatment. Van Doren et al. [41] (#3) did the same, but adapted the WET protocol for delivery in group therapy format. Finally, Hahn et al. [42] (#4) reported on a proof-of-concept study of WET combined with CBT for Alcohol Use Disorder (AUD), delivered to female survivors of recent sexual assault. This study was unique in specifying the traumatic event (recent sexual assault) that would be the focus of exposure, compared to the usual protocol where participants may select any traumatic event that they consider most distressing. Finally, Schacht et al. [43] (#6) conducted a feasibility RCT comparing outcomes from treatment as usual (TAU) in residential SUD care versus when TAU is combined with WET. Thus far, this is the only study to use a comparator group.

Four studies (#7–10) reported qualitative findings, and one quantitative study (#4) included a blended case example. The pilot conducted by Nillni et al. [38] was part of a larger project that also reported interview data gathered from WET providers [44] (#7) and users [45] (#8). Two studies used case studies to understand the experience of participants receiving WET, namely Schumacher et al., [46] (#9) who delivered WET in a bi-weekly group therapy format in residential SUD care, and Hahn et al. [47] (#10) who delivered WET in conjunction with coping skills for Cannabis Use Disorder (CUD) in female survivors of recent sexual assault.

Finally, two studies were protocols and methodologies for future pilot RCTs. Hahn et al. [48] (#11) plan to build on their pilot with WET combined with CBT for AUD, comparing its efficacy against WET alone for female survivors of sexual assault. Meshberg-Cohen et al. [49] (#12) plan to evaluate with WET combined with TAU for SUD is superior to neutral topic writing combined with TAU with a sample of veterans.

Overall, 75% of studies integrated WET into existing SUD treatments (inpatient and outpatient), while the remaining 25% were based at study clinics. In terms of population, 41.7% of study samples were women only, 25% were veterans, and 25% were patients in residential SUD treatment. Only one study (8.3%) gathered perspectives from WET providers. With regards to primary substance(s) used by participants, 75% of studies used a broad inclusion criteria allowing any or poly-substance use. Most studies applied the standard WET protocol delivered to individuals. Two studies (#3, 9) tested an adapted bi-weekly delivery of WET. Finally, an additional three studies (#4,10,11) integrated or plan to integrate WET with CBT-based interventions for AUD or CUD.

### Risk of bias assessment

Results from the RoB assessments are displayed in Table 2. Of the 5 non-randomised studies evaluated using the ROBINS-I-V2, four (#1–2, 4–5) were at moderate risk of bias and one (#3) was at serious risk. The pilot RCT (#6) assessed using the RoB 2 was at low risk of bias. Thus overall, 16.7% are at low risk of bias, 66.7% are at moderate risk, and 16.7% are at serious risk. Some sources of bias included participant selection into analysis (#3), protocol deviation (#2), and missing data (#3).

**Table 2:**
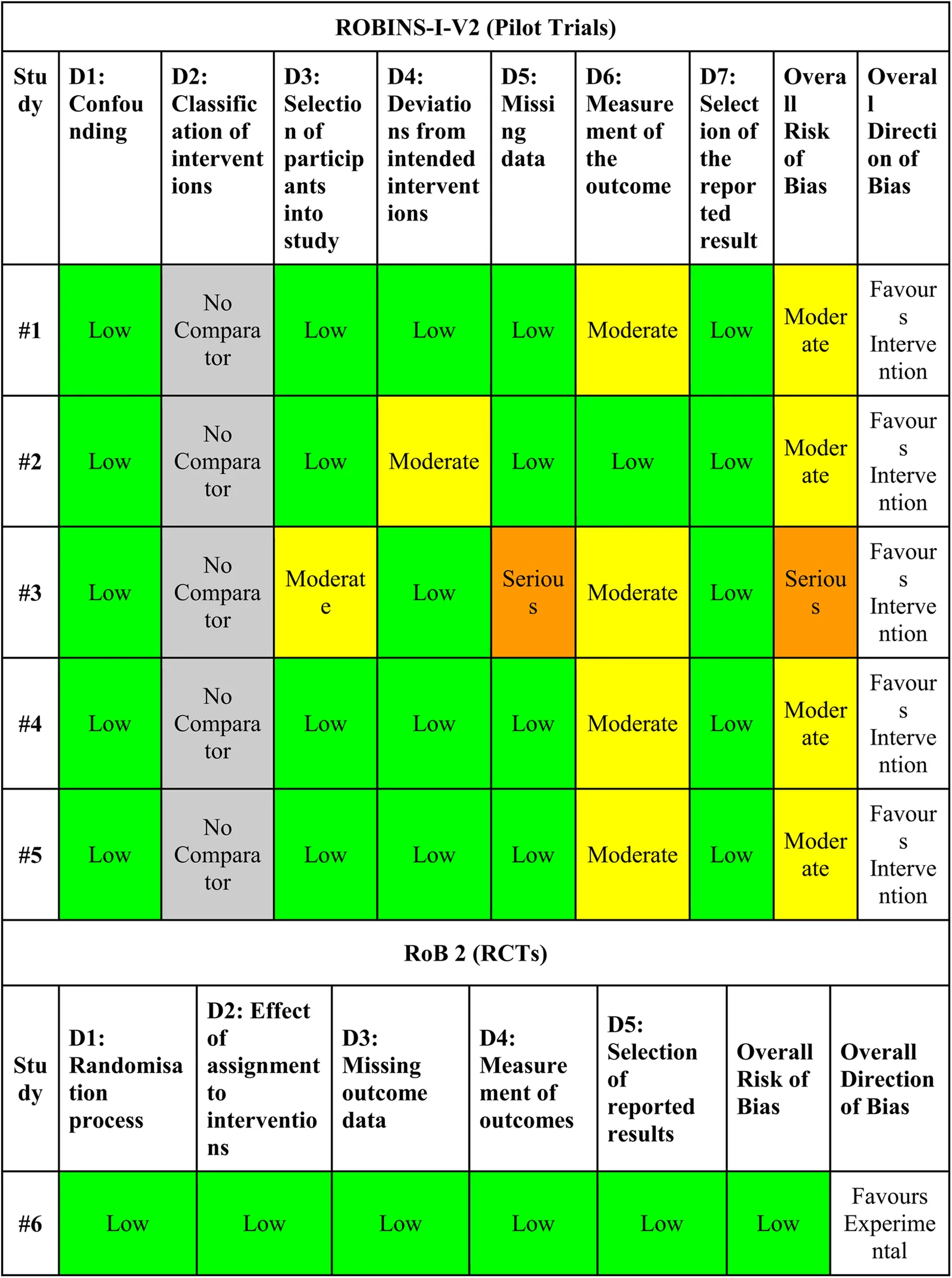
Results from Risk of Bias assessments using ROBINS-I-V2 tool for non-randomised single-arm pilot studies (#1–5) and RoB 2 tool used for pilot RCT studies (#6)

The most common source of bias in non-randomised pilots were unblinded treatment conditions (#1, 3–5), which is difficult to avoid in their design. However, even the pilot RCT (#6), despite taking some measures, was ultimately unable to achieve blinding as the comparison group (receiving only TAU) would inevitably become aware that they were the ‘control’. All studies favoured the intervention or experimental condition, possibly influenced by the awareness of receiving treatment. All studies acknowledge potential bias and limitations inherent in their design. None of the studies were biased in selection and reporting of results. These findings suggest that these studies offer valuable and foundational insights for future studies, though their results must be interpreted with caution.

### Change in PTSD symptoms

All studies used valid, reliable, and appropriate tools to establish the presence of and measure change in PTSD severity, most commonly the PCL-5. Some studies used variations of the PCL-5 and provided rationale for their use. Two studies (#3, 5) used the Weekly version as symptoms were assessed at each session; this approach is in line with the WET protocol. Two studies (#4, 10) used the Past-Week version, as they included participants whose traumatic exposure occurred less than 1 month ago.

Two studies (#2, 6) administered the Trauma Coping Self-Efficacy scale (CSE-T) [50] as an additional trauma-related instrument, and both report statistically significant improvements following WET. A pilot study, using pre/post scores (#2) reported a large effect size for increased coping efficacy (CSE-T: *t*[25] = − 5.79, *p* < 0.001, *d* = − 1.14). Similarly, a pilot RCT (#6), comparing improvement on the CSE-T for participants receiving TAU + WET versus TAU only reported significantly greater improvements among participants receiving TAU + WET (CSE-T: *M*_diff_ = 12.46, SE = 2.50, *p* < 0.001, 95% CI [− 17.51, − 7.40]), while the TAU group did not.

#### Meta-analysis measuring change in PTSD severity

**Fig. 2:**
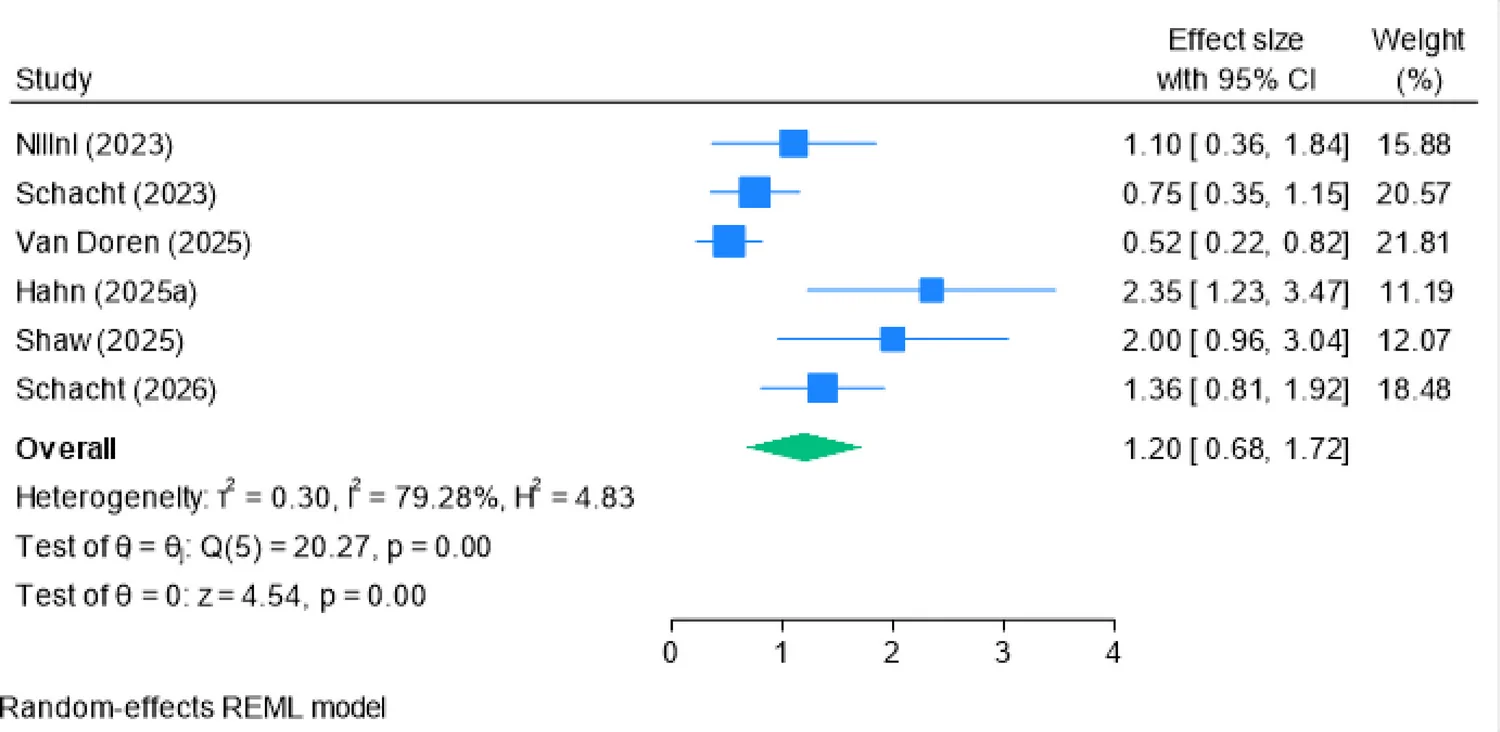
Results of random-effects meta-analysis describing change in PTSD severity as evidenced by reduction in PCL-5 (PTSD Symptom Checklist for DSM-5) scores

A random-effects meta-analysis identified a large overall pre/post effect across all six studies (Hedges’ *g* = 1.18, 95% CI [0.68, 1.72], *p* < 0.001). Effect sizes ranged from 0.52 to 2.35, indicating consistent improvement across studies. The largest effect sizes were observed for studies #4 (2.35) and #5 (2.00) respectively. The smallest effect size (0.52) is observed for study #3, which implemented WET in group format. Substantial heterogeneity was observed across studies (*I*^2^ = 79.3%), suggesting considerable variability in the magnitude of treatment-related improvement between studies. The between-study variance was moderate (τ^2^ = 0.30) and the Q test for heterogeneity was statistically significant (Q = 20.27, *p* = 0.001), indicating that the observed variability in effect sizes was greater than expected by sampling error alone. Results of the meta-analysis including effect sizes are presented in Table 3, and the forest plot for included studies is presented in Fig. 2.

**Table 3:** Results from a random-effects meta-analysis (REML) for reduction in PTSD severity as measures by change in PCL-5 scores

Study	Effect size	[95% conf. interval]		% weight
Nillni (2023)	1.102	0.363	1.842	15.88
Schacht (2023)	0.751	0.348	1.154	20.57
Van Doren (2025)	0.518	0.221	0.815	21.81
Hahn (2025 a)	2.347	1.227	3.466	11.19
Shaw (2025)	1.997	0.958	3.036	12.07
Schacht (2026)	1.362	0.805	1.919	18.48
theta	1.198	0.680	1.715	

Test of theta = 0: z = 4.54 Prob > |z| = 0.0000

Test of homogeneity: Q = chi2(5) = 20.27 Prob > Q = 0.0011

**Fig. 3:**
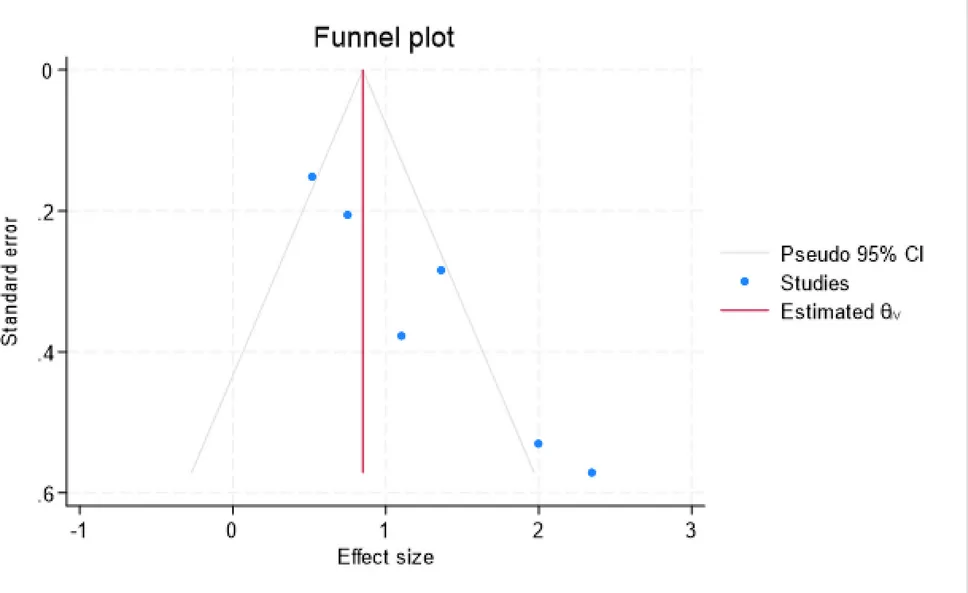
Funnel plot describing the results of Egger’s regression test used to detect small-study effects and publication bias for studies included in the meta-analysis

Visual inspection of the funnel plot (see Fig. 3) suggested possible asymmetry, with smaller studies tending to report larger effect sizes. Egger’s regression test was statistically significant (*β* = 3.99, SE = 0.93SE, *z* = 4.28, p < 0.001), indicating potential small-study effects. However, these findings should be interpreted cautiously due to the small number of included studies and the substantial between-study heterogeneity, both of which limit the reliability of publication bias assessments. In addition, because most included studies used uncontrolled pre/post designs, the pooled effect reflects symptom improvement over time among participants receiving WET and does not provide definitive evidence of treatment efficacy relative to control conditions.

#### Key learnings from qualitative data

Change in PTSD symptoms was reported through the experience of seven participants across different studies (#4, 9–10), six females and one male. A significant reduction in PTSD symptoms was noted for most participants, as measured by PCL-5 and/or CAPS-5 scores. Case studies differ in the data they reported, e.g., some reported PCL-5 scores while others reported distress before and after written exposure.

One case series (#9) reported a reduction of 69% or more in PCL-5 scores for *n* = 3 participants. Two studies (#4, 10) reported Subjective Units of Distress (SUDs) pre- and post-writing for *n* = 4 participants. In the first session, average distress increased from 72.5/100 pre-writing to 87.5/100 post-writing. As treatment progressed, two participants reported increased SUDs post writing, one participant reported decreased SUDs post-writing, and one participant reported no change in SUDs from pre- to post-writing. Despite individual variance, by session 5 average SUDs had decreased to 35/100 pre-writing and 40/100 post-writing. Participants benefited from therapeutic support and emotion regulation tools to continue writing and remain engaged in treatment.

Individual variability was noted in how PTSD symptoms reduced over time, with sharp declines for some participants and steady reduction for others. Some participants ended therapy in less than five sessions as they had achieved a satisfactory outcome, while others required 1–3 additional sessions. Although most participants reported a reduction in PTSD severity, not all achieved remission of symptoms. Participants with complex, lifetime histories of trauma reported a smaller reduction in PTSD severity. One possible explanation is that their symptoms were associated with multiple traumatic experiences rather than a single identifiable event, although this was not directly examined in the present data.

### Change in SUD symptoms

Six studies (#1, 3, 5–6, 8–9) assumed the presence of SUD; it can be safely presumed that the SUD treatment facilities they are based in had existing screening and diagnostic procedures in place. Two studies (#2, 10) used the diagnostic Mini-International Neuropsychiatric Interview [51] and one study (#4) used the AUD Identification Test (AUDIT-C) [52] as a screening tool.

Two studies reported change in alcohol use (#4) and cannabis use (#10) using the Timeline Follow-Back [53]. Study #4 reported a statistically significant 37.4% decrease in drinking days, with 1.62 fewer standard drinks consumed on drinking days, and 4.89% fewer days of heavy drinking at 1-month follow-up compared to baseline. Study #9 reported outcomes for cannabis use for three participants in a case series. Cannabis use ranged from 23.3–100% at baseline, 3.3–63.3% at post-treatment, and 3.3–26.7% at 1-month follow-up. For 2 of the 3 participants, cannabis use did not decrease further from post-treatment to follow-up.

Further four studies did not measure change in substance use directly, and instead measured related factors that impact recovery. One study (#1) used a single item from the 3-item Craving Scale [54] and reported a medium reduction 1.5 in craving intensity from baseline to follow-up. One case series (#9) implemented the Drinking Refusal Self-Efficacy Questionnaire [55] but did not report results. Two studies (#2, 6) used the Brief Assessment of Recovery Capital [56] to measure internal, social, and environmental resources facilitating recovery. The first was a pilot study (#2) that reported statistically significant increase in participants’ recovery capital (*t*[25] = − 4.24, *p* < 0.001, *d* = − 0.83). The second was a pilot RCT (#6) that compared scores for participants receiving TAU + WET versus TAU alone. In pairwise comparisons by condition, both groups showed statistically significant improvements in recovery capital, with higher post-treatment improvement for the TAU + WET group (*M*_diff_ = 5.40, SE = 2.57, *p* < 0.001, 95% CI [− 10.60, − 0.20]). In pairwise comparisons by time, the TAU + WET group showed statistically significant improvement in recovery capital (*M*_diff_ = 7.84, SE = 1.75, *p* < 0.001, 95% CI [− 11.37, − 4.31]) while the TAU group did not. There was a significant condition x time interaction (*F*(1, 40) = 4.33, *p* = 0.001, n^2^_p_ = 0.10), suggesting that increase in recovery capital over time was dependent on the treatment condition.

#### Key learnings from qualitative data

Case studies (#4, 9–10) reported data from seven participants (six female and one male). One study (#9) did not provide data on change in substance use. Two studies (#4, 10) reported craving intensity for primary substance used pre- and post- written exposure for *n* = 4 participants. In the first WET session, the average craving intensity increased from 55/100 pre-writing to 77.5/100 post-writing. However, overall craving intensity subsequently declined, such that by session three average craving was 37.5/100 pre-writing and 40/100 post-writing. By session five, craving intensity was 0/100 for three participants and 70/100 for the fourth participant, with no post-writing change observed.

### Provider training and treatment fidelity

Most studies (#1–3, 5–8) provided regular clinical supervision and case consultations to foment provider training and treatment fidelity. Three of these studies (#1, 7–8) report on the results of the same pilot, whose providers were trained to fidelity by completing a 5-h pre-recorded presentation by a co-developer of WET followed by a 2-h in-person training delivered by study authors. These studies (#1, 7–8) utilised session recordings rated for fidelity by non-study therapists. Providers received mean adherence scores of 95.9% for adherence to the treatment manual and a competence score of 6.7 out of 7.0 for treatment delivery (reported in study #1). In qualitative feedback (reported in studies #7–8), providers note that the extent of supervisory support during the pilot was higher than is typical for routine treatment settings. They voiced concern that a reduction in supervisory support as the treatment is scaled up may reduce fidelity.

Two studies (#2, 6) utilised only the 2-h in-person training component of WET to train providers. One of these (#2) did not assess for fidelity, while the other (#6) required providers to submit session tracking sheets after each session to be assessed for treatment adherence. One study (#3) utilised a provider who had previous training in WET and monitored fidelity through provider self-checks and regular consultation of the WET manual. Another study (#5) utilised providers with existing expertise in PTSD treatment and did not provide specific training for WET, stating that the straightforward manual would be well-understood upon reading. Three studies (#4, 9–10) and one planned RCT (#12) provide no information regarding provider training or treatment fidelity. Finally, one planned RCT (#11) reported plans to use session recordings to evaluate fidelity.

### Feasibility and retention

A common way to examine the feasibility of a treatment is through participant retention. Table 4 details participant retention for studies #1–6 as they had adequate samples. For this review, enrollment is defined as attending at least 1 session of WET, and retention is defined as attending at least 5 sessions, which ensures completion of the WET protocol. Enrolled participants may have attended between 1–4 sessions, and retained participants may have completed more than 5. A more granular detailed analysis of session attendance was beyond the scope of this review. From the 201 (Range: 11–63) participants eligible for WET, 155 (Range: 10–48; 77.11%) were enrolled and 107 (Range: 10–30; 53.23%) were retained. From 155 enrolled participants, 69.03% were retained. Dropout was related to avoidance, logistics (competing life/medical needs), and extraneous variables (a storm disrupting treatment). Demographic and clinical characteristics were not related to dropout, nor were the number/types of traumatic exposures. Psychoeducation about PTSD-SUD, normalising increased distress after exposure, and emotion regulation tools supported retention.

**Table 4:** Participant retention for WET showing number of participants who were eligible, who completed at least 1 treatment session, and who completed at least 5 treatment sessions

**Study**	**Eligible**	**Enrolled** (Completed at least 1 session)	**Retention** (Completed at least 5 sessions)
#1	42	18	10
#2	49	45	30
#3	63	48	28
#4	11	11	10
#5	11	10	10
#6	25	23	19
Total (*N*)	201	155	107

Number of studies = 6

Heterogeneity:

tau2 = 0.2966

I2 (%) = 79.28

H2 = 4.83

Two studies (#2, 6) used the Barriers to Treatment Participant Scale [57] to gauge feasibility, where a lower score indicates fewer barriers. From a possible range of 23–115, study participants gave WET a mean score of 36.44 (SD = 18.16) and 31.42 (SD = 10.31) respectively. WET proved adaptable and flexible, as it was successfully delivered in diverse settings. Notably, it was feasible in a low-resource, high-burden residential care settings where previous attempts to integrate prolonged exposure had failed (#5). It was successfully adapted to a twice-weekly group therapy format (#3, 9), showing potential for an even more brief and efficient delivery. The delivery of WET via telehealth was feasible (#7), but created therapeutic barriers as patients more readily avoided exposure through distraction or had limited privacy and safety.

Providers also rated WET as a feasible treatment. In survey responses (#7), providers rated the usefulness of WET as 81.8/100 (SD = 20.4), its helpfulness as 4.40/5 (SD = 0.84), and its appropriateness as 97.5/100 (SD = 7.0). WET was considered an appropriate treatment for 86% of patients with PTSD-SUD. Its brevity and ease of integration were considered advantageous. Most providers (*M* = 98.6/100, SD = 5.3) intended to continue using WET, but voiced the need for training and supervision to help adoption and sustainability.

### Acceptability

WET was shown to be an acceptable treatment across multiple studies. Two studies (#1, 4) implemented the Client Satisfaction Questionnaire [58] and reported similar mean scores of 30.33 (SD = 1.88) and 30.3 (SD = 2.4) out of a maximum score of 32. Participant’s trust in the treatment increased over time, evidenced by the Credibility/Expectancy Questionnaire (CEQ) [59] used by two studies (#4, 6). Notably, in a pilot RCT (#6), mean CEQ scores increased from 42.9 at baseline to 43.8 at follow-up for those receiving TAU + WET, but decreased from 41.0 to 38.5 for those receiving TAU only. Finally, WET was also ranked highly on the Acceptability of Intervention Measure [60] implemented in study #4; it received a mean score of 16.86/20 (SD = 2.79).

Based on a synthesis of qualitative data reported across almost all studies regarding patient experience, a majority of participants found WET an acceptable treatment. 100% of participants across studies said they would recommend WET to future patients. The majority of participants were open to integrated treatment and found it helpful, particularly as some of them faced SUD-related discrimination as a barrier to receiving PTSD treatment. A minority of participants found addressing PTSD and SUD concurrently to be too burdensome and did not complete the WET protocol.

Finally, WET was acceptable to treatment providers. Responding to survey and interview questions (#5, 8), providers reported that WET was not burdensome, was helpful to their patients, and that they would recommend it to future patients. Practitioners foresaw themselves using WET as an ongoing treatment modality in their clinical work.

## Conclusions

The aim of this study was to evaluate the efficacy, feasibility, and acceptability of Written Exposure Therapy (WET) as a component of integrated treatment for comorbid PTSD-SUD. 12 studies were included in the review: 5 were single-arm pilots, 4 were qualitative studies, 1 was a pilot RCT, and 2 were pilot RCTs pending implementation. Results show that WET was effective in reducing PTSD severity, and was feasible and acceptable to both participants and providers.

A random-effects meta-analysis of six studies demonstrated that WET contributed to significant reductions in PTSD severity, as evidenced by PCL-5 scores. However, substantial heterogeneity in effect sizes suggests that factors such as study design, treatment setting, or variations in SUD interventions may influence change in PTSD severity. The most pronounced reduction in PTSD was observed in study #4, which combined WET with CBT for AUD among survivors of recent sexual assault, all of whom wrote about their recent trauma as the focal index event for exposure. Our findings are consistent with existing evidence that WET can be less effective in reducing trauma-related beliefs when more time has elapsed since the index trauma [61], indicating that focusing on recent trauma may yield greater reductions in PTSD severity.

All participants reported declines in PTSD severity, though not all achieved remission of symptoms. The standard 5-session WET protocol was adequate for most participants, while some required additional sessions to maximise symptom reduction. Those with complex, lifetime trauma histories reported more limited improvement and were referred to additional treatment. This implies that WET may serve as a gateway therapy for individuals with complex trauma, facilitating acclimation to exposure before pursuing more intensive exposure treatments.

Average retention in WET across all quantitative studies (#1–6) was 69.0%, compared with reported retention rates ranging from 32 to 61% for integrated Prolonged Exposure therapy for PTSD-SUD [25, 62–67]. It was also successful (though least effective) when adapted to a twice-weekly group format (#3, 9). Existing evidence supports the use of such accelerated, massed treatment protocols for the exposure-based treatment of PTSD as they are non-inferior to standard protocols in reducing PTSD severity, and can improve retention and treatment engagement by reducing avoidance and between-session distractions [68–73]. Ennis et al. [74] implemented a massed protocol for WET on an inpatient mental health unit; they reported significant reduction in PTSD severity, low dropout (16.7%), and acceptability to treatment recipients. Pruiksma et al. [75] delivered massed WET in a workshop-style group format and reported similarly positive outcomes for PTSD severity and treatment acceptability. These data suggest that WET has strong potential utility in high-burden, low-resource treatment settings.

Finally, WET was an acceptable treatment for the majority of providers and recipients. A minority of participants (#8) reported that they found concurrent treatment of PTSD and SUD burdensome, suggesting that some individuals may prefer sequential treatment. Finally, providers found integrated WET an acceptable and feasible treatment modality, and the majority intended to continue using WET in their practice.

### Limitations and future directions

This study generated foundational knowledge about the use of WET as a component in integrated treatment for PTSD-SUD through a systematised process. Meta-analysis pooled existing evidence regarding the effectiveness of WET in reducing PTSD severity. However, several limitations should be considered. 5 of the 6 studies assessed were at moderate (#1–2, 4–5) or serious (#3) risk of bias. Common methodological limitations include small samples, lack of blinding, and predominance of uncontrolled pre/post designs. Studies varied in how they trained providers to deliver WET or did not comment on provider training. Most studies used regular clinical supervision to monitor treatment fidelity, but few used more structured methods (e.g., review of session recordings) to measure fidelity. Variation in treatment fidelity may partially explain heterogeneity observed in the current findings. Overall, these factors limit the reliability, generalisability, and causal interpretation of findings.

Considerable heterogeneity was also observed across studies in treatment settings, participant characteristics, study design, and the nature of concurrent SUD interventions. In this review, 50% of studies were situated in residential SUD care, a setting associated with higher rates of retention and treatment completion compared to outpatient SUD treatment [76, 77]. This may have contributed to the higher retention rates seen in this review. In several studies, WET was delivered alongside multi-component SUD treatment programs, making it difficult to determine which aspects of integrated care contributed most strongly to treatment outcomes. Several studies did not measure change in SUD directly or at all, and reductions dependence cannot be conclusively inferred without direct measurement.

Future studies should prioritise adequately powered RCTs with clearly defined comparator conditions. Comparing the effectiveness of WET to neutral writing exercises (as is planned by study #12) will address existing limitations due to lack of blinding. Future studies should provide more standardised descriptions of concurrent SUD treatments, and additionally explore the acceptability and feasibility of WET in outpatient services. Robust fidelity monitoring and more standardised provider training should be prioritised to ensure consistent effectiveness across diverse settings and to facilitate the translation into routine clinical practice. Finally, future studies should utilise larger and more diverse samples as well as longer follow-up periods. These efforts will help clarify the effectiveness of WET within integrated PTSD-SUD treatments and determine its optimal role in clinical practice.

## Key References

- Sloan DM, Marx BP. Written Exposure Therapy for PTSD: A Brief Treatment Approach for Mental Health Professionals. 2nd ed. Washington (DC): American Psychological Association; 2025.
  - ○ This reference is important as it is the most recent published manual of Written Exposure Therapy (WET). It details supporting literature, mechanisms of change in WET, and manualised protocol of administering WET.
- Hahn CK, Tilstra-Ferrell EL, Salim SR, et al. Written Exposure Therapy Integrated With Cognitive Behavioral Skills for Alcohol Misuse: A Proof-of-Concept Study With Recent Sexual Assault Survivors. Cognitive and behavioral practice. 2026;33(2):231–245.https://doi.org/10.1016/j.cbpra.2024.12.005.
  - ○ This reference is a recent study of WET integrated with CBT for AUD as treatment for comorbid PTSD-AUD. In the meta-analysis performed in this study, this study reported the largest effect size for improvement in PTSD severity.
- Shaw MT, Schmidt JLD, Lehman PK, Holohan DR. Written Exposure Therapy for Dually Diagnosed Veterans in a Residential Domiciliary Substance Use Treatment Program: A Pilot Study. Traumatology (Tallahassee, Fla). Published online September 4, 2025. https://doi.org/10.1037/trm0000628.
  - ○ This reference is a recent study of WET integrated in a residential SUD treatment setting. This study shows the second-largest effect size for improvement in PTSD severity.
- Schacht RL, Wenzel KR, Meyer LE, Berg SK, Fishman M. Written exposure therapy for post-traumatic stress disorder in residential treatment for substance use disorders: A randomized feasibility trial. Addictive behaviors. 2026;180:108732. https://doi.org/10.1016/j.addbeh.2026.108732.
  - ○ This reference is the first feasibility RCT of integrated WET for the treatment of comorbid PTSD-SUD. So far, it is the only study to use a comparator group, and offers valuable insights as it uses a comparator group.
- Valentine SE, Godfrey LB, Gellatly R, et al. Supporting the implementation of written exposure therapy for posttraumatic stress disorder in an obstetrics-substance use disorder clinic in the Northeastern United States. SSM - mental health. 2023;4:100256. https://doi.org/10.1016/j.ssmmh.2023.100256.
  - ○ This reference is important as it is the only study to gather perspectives from providers of WET regarding its implementation, feasibility, and acceptability as a treatment for comorbid PTSD-SUD.

## Supplementary Information

Supplementary Material 1.

## Data Availability

No datasets were generated or analysed during the current study.
