# Supplementary material for: Written exposure therapy as treatment for comorbid PTSD and SUD: a systematic review and meta-analysis
Source: Curr Psychiatry Rep. 2026 Nov 7;28(1):75. doi: 10.1007/s11920-026-01724-x (PMC13743590; doi:10.1007/s11920-026-01724-x)
Supplement: Supplementary file 1 [file 11920_2026_1724_MOESM1_ESM.docx]

**Appendix A**

The following inclusion criteria were used for the systematic review:

a)     Publications in the English language.

b)    Studies that implement or plan to implement Written Exposure Therapy (WET) as a treatment for adults presenting with co-morbid PTSD/trauma and substance use disorder.

c)     Presence of co-morbid diagnoses or symptoms of both trauma and substance use must be established for treatment participants.

d)    Participants may meet diagnostic criteria for PTSD or have been otherwise assessed to have elevated PTS symptoms using reliable instruments such as the Clinician-Administered PTSD Checklist for DSM-5 (CAPS-5), and/or the Life Events Checklist for DSM-5 (LEC-5) and/or the Post-Traumatic Disorder Checklist for DSM-5 (PCL-5).

e)     Participants may meet diagnostic criteria for any substance use disorder as established by use of a reliable instrument such as the Alcohol Use Disorders Identification Test (AUDIT-C), or established in the context of participants in a residential, outpatient, or other treatment/rehabilitation program for substance use. Substance of choice may be alcohol, other drugs, or polysubstance use.

f)     Studies that use quantitative and/or qualitative methods, as well as published protocols for future studies. Studies may gather data from recipients or practitioners of WET in the context of its use as a treatment for co-morbid trauma and substance use.

g)    Studies may compare WET with a comparator treatment, control group, or have no comparator/control group.

Searches were performed in major databases including PsycINFO, PsycArticles, Wiley Online, Pilots, PubMed, Scopus, and Monash Library Database. The following search terms were combined to generate relevant results: “written exposure therapy” [AND] “PTSD”, “trauma” [AND] “substance use disorder”, “alcohol and other drugs”, “alcohol”, “marijuana”, “opioids”, “narcotics”, “benzodiazepines”, “methamphetamine OR amphetamine”, “stimulants”, “cocaine”, “barbiturates”, “ecstasy”, “y-hydroxybutyric acid (GHB)”, “hallucinogen”, “heroin”, “3, 4 methylenedioxy methamphetamine (MDMA)”, “oxycodone”, “polysubstance”, “methadone”.
